# Association of Neonatal Pain-Related Stress and Parent Interaction With Internalizing Behaviors Across 1.5, 3.0, 4.5, and 8.0 Years in Children Born Very Preterm

**DOI:** 10.1001/jamanetworkopen.2022.38088

**Published:** 2022-10-21

**Authors:** Mia A. McLean, Olivia C. Scoten, Cecil M. Y. Chau, Anne Synnes, Steven P. Miller, Ruth E. Grunau

**Affiliations:** 1 BC Children’s Hospital Research Institute, Vancouver, British Columbia, Canada; 2 Department of Pediatrics, University of British Columbia, Vancouver, British Columbia, Canada; 3 BC Women’s Hospital, Vancouver, British Columbia, Canada; 4 Department of Neurology, The Hospital for Sick Children, Toronto, Ontario, Canada; 5 Department of Paediatrics, University of Toronto, Toronto, Ontario, Canada

## Abstract

**Importance:**

Internalizing (anxiety and/or depressive) behaviors are prevalent in children born very preterm (24-32 weeks’ gestation). Procedural pain-related stress in the neonatal intensive care unit (NICU) is associated with long-term internalizing problems in this population; however, whether positive parenting during toddlerhood attenuates development of internalizing behaviors across childhood is unknown.

**Objective:**

To investigate whether neonatal pain-related stress is associated with trajectories of internalizing behaviors across 1.5, 3.0, 4.5, and 8.0 years, and whether supportive parenting behaviors and lower parenting stress at 1.5 and 3.0 years attenuate this association.

**Design, Setting, and Participants:**

In this prospective longitudinal cohort study, preterm neonates (born at 24-32 weeks’ gestation) were recruited from August 16, 2006, to September 9, 2013, with follow-up visits at ages 1.5, 3.0, 4.5, and 8.0 years. The study was conducted at BC Women’s Hospital, Vancouver, Canada, with recruitment from a level III neonatal intensive care unit and sequential developmental assessments performed in a Neonatal Follow-up Program. Data analysis was performed from August to December 2021.

**Main Outcomes and Measures:**

Parental report of child internalizing behaviors on the Child Behavior Checklist at 1.5, 3.0, 4.5, and 8.0 years.

**Results:**

A total of 234 neonates were recruited, and 186 children (101 boys [54%]) were included in the current study across ages 1.5 (159 children), 3.0 (169 children), 4.5 (162 children), and 8.0 (153 children) years. After accounting for clinical factors associated with prematurity, greater neonatal pain-related stress was associated with more internalizing behaviors across ages (B = 4.95; 95% CI, 0.76 to 9.14). Higher parenting stress at age 1.5 years (B = 0.17; 95% CI, 0.11 to 0.23) and a less supportive parent environment (less sensitivity, structure, nonintrusiveness, nonhostility, and higher parenting stress; B = −5.47; 95% CI, −9.44 to −1.51) at 3.0 years were associated with greater internalizing problems across development to age 8.0 years.

**Conclusions and Relevance:**

In this cohort study of children born very preterm, exposure to repetitive neonatal pain-related stress was associated with persistent internalizing behavior problems across toddlerhood to age 8.0 years. Supportive parenting behaviors during early childhood were associated with better long-term behavioral outcomes, whereas elevated parenting stress was associated with more child anxiety and/or depressive behaviors in this population. These findings reinforce the need to prevent pain in preterm neonates and inform future development of targeted parent-led behavioral interventions.

## Key Points

**Question:**

Does supportive parenting ameliorate the association between neonatal pain-related stress and child internalizing behaviors in children born very preterm?

**Findings:**

In this cohort study of 186 children born very preterm, internalizing behaviors increased across ages 1.5, 3.0, 4.5, and 8.0 years, and more neonatal pain-related stress was associated with greater internalizing behaviors across ages. At 1.5 years, parenting stress was associated with more internalizing behaviors, whereas at age 3.0 years, a more supportive parenting environment was associated with fewer internalizing behaviors across development.

**Meaning:**

These findings suggest that supportive parenting is associated with reduced child anxiety and depressive behaviors from toddlerhood through school-age in children born very preterm.

## Teaser

This cohort study investigates whether neonatal pain-related stress is associated with trajectories of internalizing behaviors across 1.5, 3.0, 4.5, and 8.0 years, and whether supportive parenting behaviors and lower parenting stress at 1.5 and 3.0 years attenuate this association.

## Introduction

Internalizing behavior problems (anxiety and/or depressive symptoms) are prevalent in children born preterm (<37 weeks’ gestation).^1,2,3,4,5^ Symptoms are evident as early as toddlerhood.^6,7,8,9^ Those who show more internalizing behaviors in early childhood are likely to show long-term problems through adolescence,^3^ often with increases in symptoms^10,11^ impacting social and school functioning, family, and community.^12^

As neonates, infants born very preterm (24-32 weeks’ gestation) spend their first weeks to months of life in the neonatal intensive care unit (NICU). During a period of neurobiological vulnerability, they are exposed to the stress and pain of frequent invasive procedures.^13,14,15,16,17^ Beyond adverse clinical factors related to prematurity (eg, illness severity at NICU admission, analgesics, and infection), we have previously shown that NICU procedural pain-related stress is associated with greater internalizing behaviors at 1.5 years^9^ and at school age.^18^ Moreover, considerable literature suggests that supportive and structured parenting is associated with improved neurodevelopmental and behavioral outcomes in children born very preterm^19,20^; however, few longitudinal studies have investigated long-term benefits.^6,21,22^ In an earlier cohort,^9^ we found that sensitive and warm (nonhostile) parent interactions partially ameliorated the negative association of neonatal pain-related stress on internalizing behaviors at age 1.5 years, whereas parenting stress was associated with greater prevalence of these behaviors. It is unknown whether a supportive parenting environment (parenting stress and parent behavior) attenuates the association between neonatal pain-related stress and child internalizing behaviors beyond 1.5 years.

In the present study, in an independent cohort of children born very preterm, we addressed the hypotheses that (1) greater neonatal pain-related stress is associated with more internalizing behaviors across ages 1.5, 3.0, 4.5, and 8.0 years; (2) multiple aspects of the parenting environment co-occur, such that a supportive parenting environment would encompass low parenting stress, greater sensitivity, nonintrusiveness, greater structuring, and nonhostile parent behaviors; and (3) a more supportive parenting environment at ages 1.5 and 3.0 years attenuates the negative association of neonatal pain-related stress with internalizing behaviors across development.

## Methods

The University of British Columbia and BC Women’s Hospital Clinical Research Ethics Board approved this study. Parental written informed consent was obtained at study enrollment and again at each follow-up visit. This study follows the Strengthening the Reporting of Observational Studies in Epidemiology (STROBE) reporting guideline for cohort studies.

### Study Population

In a prospective longitudinal cohort study, infants born very preterm (24-32 weeks’ gestational age) were recruited from the level III NICU at BC Women’s Hospital in Vancouver Canada from August 6, 2006, to September 9, 2013. Inclusion criteria included being born at 24 to 32 weeks’ gestational age; no major congenital malformation or syndrome; no antenatal toxoplasmosis, cytomegalovirus, rubella, or herpes virus infection; and/or no large periventricular hemorrhagic infarction (>2 cm) on clinical ultrasonography (eFigure 1 and eAppendix 1 in the Supplement).

### Participant Demographics

Parent(s) reported on demographics including maternal and paternal education level and years, age, and ethnicity. Ethnic background options (ie, Black, East/Southeast Asian, East Indian, Filipino, First Nations, White, and any other) were defined by investigators and were assessed in this study to provide detailed demographic information about the study sample.

### Neonatal Clinical Factors

Prospective daily medical and nursing medical record reviews were conducted by neonatal research nurses to obtain data on clinical factors from birth to NICU discharge or term-equivalent age, whichever came first (Table 1). Clinical factors were selected to be consistent with our prior work examining pain and/or stress, parenting, and child behavior.^9^ Cumulative pain and/or stress was defined as the number of invasive procedures from birth to NICU discharge or term equivalent age, whichever came first, as in our previous studies.^23^

**Table 1.** Neonatal Clinical Factors

Variable	Mean (SD)	Median (IQR) [range]
Gestational age at birth, wk	28.0 (2.2)	27.71 (26.0-29.9) [24.0-32.3]
Sex, neonates, No. (%)		
Male	101 (54)	NA
Female	85 (46)	NA
Total time in neonatal intensive care unit, d	62 (47)	48 (20-101) [3-221]
Neonatal pain-related stress, No. of invasive procedures from birth to term	120.6 (78.9)	96.0 (64.0-168.0) [14.0-385.0]
Score for Neonatal Acute Physiology–II	12.7 (12.8)	9.0 (0.0-17.0) [0.0-57.0]
Morphine exposure, cumulative dose adjusted for daily body weight, mg	4.3 (10.3)	0.1 (0.0-2.7) [0.0-58.3]
Time receiving mechanical ventilation and/or oscillation, d	48.8 (36.4)	44.0 (12.0-84.0) [0.0-111.0]
Surgical procedures, No. (%)		
0	134 (72)	NA
1	30 (16)	NA
2-4	22 (12)	NA
Culture-confirmed postnatal infection, No. (%)	89 (48)	NA

Abbreviation: NA, not applicable.

### Parenting Stress

At ages 1.5 and 3.0 years, caregivers reported on their level of parenting stress using the Parenting Stress Index (PSI).^24^ The PSI is a 120-item questionnaire, for which each item was rated by parents via a 6-point Likert scale from 1 (strongly agree) to 6 (strongly disagree). We used the total raw score for the Parent Domain in the current study, which sums scores on 54 items across 7 subscales measuring stress related to parenting characteristics: Competence (13 items), Isolation (6 items), Attachment (7 items), Role Restriction (7 items), Depression (9 items), Spouse/Parenting Partner Relationship (7 items), and Health (5 items). Scores on the Parent Domain range from 54 to 270; higher scores indicate greater parenting stress.

### Parent and Child Interactive Behaviors

Parent and child interactions during a 5-minute semistructured teaching task (described in eAppendix 1 in the Supplement) were filmed and then rated separately by independent coders (1 primary and 2 reliability; not coauthors of this article) who were blinded to all information, using the Emotional Availability (EA) Scale–IV.^25^ Parent behaviors included sensitivity (appropriateness and authenticity of affect), nonhostility (nonthreatening, warm), nonintrusiveness (lack of overprotection, allows autonomy), and structuring (guidance); child behaviors included responsiveness (affect, organization of behavior, attention) and involvement (physical and verbal, initiation of play). Scores on each dimension range from 7 to 29, with scores of 26 to 29 considered optimally emotionally available, scores of 18 to 25 considered inconsistently emotionally available, and scores of 7 to 17 considered nonoptimally emotionally available per clinical cutoff criterion. Dimensions of EA have been widely used to study parent-child interaction in normative^26,27^ and preterm^9,22^ dyads. We summed child involvement and responsiveness to create an EA dimensional sum of child interactive behavior at each age.^25^ To account for child-associated parent behaviors, we controlled for child interactive behaviors when examining relationships between parent interactive behaviors and child internalizing behaviors.

### Child Internalizing Behaviors

The Child Behavior Checklist (CBCL) 1.5 to 5 years^28^ questionnaire was completed by the primary caregiver (85% mothers) at ages 1.5, 3.0, and 4.5 years; at age 8.0 years, the CBCL for years 6 to 18 was completed.^29^ Here, we used the CBCL-Internalizing (Withdrawn, Somatic Complaints, Anxious/Depressed) subscale T-score (mean [SD], 50 [10]). Higher scores represent greater problems; a T-score of 64 or higher is the clinical problem cutoff, and a score of 60 or higher is the subclinical problem cutoff.

### Statistical Analysis

 Data analysis was performed from August to December 2021. Analyses were conducted in R statistical software version 4.1.0 (R Project for Statistical Computing).^30^ Participant characteristics were compared across visit ages using analysis of variance and 2-sided χ^2^ tests, with significance set at *P* < .05. Because of nonnormal distributions, cumulative morphine exposure and neonatal pain-related stress were log-transformed, and a square-root transformation was applied to days on mechanical ventilation and/or oscillation. Pearson correlations among study variables were performed. Latent profile analysis via gaussian mixture modeling was used to identify profiles of parent environment at ages 1.5 and 3.0 years from continuous measures of parent behaviors and parenting stress via the mclust^31^ package (hypothesis 2). To examine hypotheses 1 and 3, multilevel models were conducted with time-dependent data (CBCL Internalizing at each age) nested within individuals using lme4^32^ and lmerTest^33^ R packages. Across ages, missing data were found to be random (eFigure 2 and eTable 1 in the Supplement). We optimized model fit while accounting for missing participant-level data across assessment ages using the multilevel joint modeling multiple imputation,^34^ jomo,^35^ and mitml^36^ R packages. We report estimated coefficients and SEs pooled across 100 imputed data sets for multilevel models. Likelihood ratio tests (LRTs) for multiply imputed data sets^37^ were used to compare models at each stage to establish the most parsimonious explanatory model. See eAppendix 2 in the Supplement for further details.

## Results

### Cohort Description

A total of 234 neonates were recruited. After exclusions, 186 participants (101 boys [54%]) had valid neonatal clinical data (Table 1) and CBCL Internalizing scores at 1 or more assessment age: 159 children at 1.5 years, 169 children at 3.0 years, 162 children at 4.5 years, and 153 children at 8.0 years, with 120 (65%) having parental report of behaviors at all 4 study ages (Table 2). Demographic indicators of participants who attended did not differ across visits (Table 2). At 1.5 years, 3 children met the cutoff for clinical problems, and 24 of 153 children (16%) met this criterion at 8.0 years. See eAppendix 3 and eTable 2 in the Supplement for study variable intercorrelations. Attrition analyses are provided in eTable 3 in the Supplement.

**Table 2.** Parent and Child Characteristics at Follow-up Visits

Variable (N = 186)	1.5 y Corrected age	3.0 y	4.5 y	8.0 y
Maternal age at birth, mean (SD) [range], y	33.0 (5.2) [22.1-45.9]	32.8 (5.1) [21.8-45.9]	32.9 (5.2) [21.8-45.9]	32.6 (5.2) [21.8-45.9]
Maternal education level, mothers, No. (%)				
Primary or secondary school graduation	24 (15)	21 (13)	25 (16)	23 (15)
Partial or complete undergraduate degree	101 (64)	114 (68)	105 (66)	98 (65)
Postgraduate university degree	32 (20)	32 (19)	29 (18)	29 (19)
Parent sensitivity score				
Mean (SD) [range]	20.2 (4.0) [10.0-29.0]	21.4 (3.6) [12.5-29.0]	NA	NA
Missing, No.	29	21	NA	NA
Parent nonhostility score				
Mean (SD) [range]	21.7 (3.5) [12.0-29.0]	22.4 (3.1) [15.0-29.0]	NA	NA
Missing, No.	29	21	NA	NA
Parent nonintrusiveness score				
Mean (SD) [range]	19.1 (4.9) [8.0-29.0]	20.7 (4.4) [10.0-29.0]	NA	NA
Missing, No.	29	21	NA	NA
Parent structuring score				
Mean (SD) [range]	20.9 (3.4) [13.0-28.5]	22.4 (3.6) [13.5-29.0]	NA	NA
Missing, No.	29	21	NA	NA
Child interactive behavior score				
Mean (SD) [range]	38.3 (6.4) [14.0-55.5]	41.3 (6.5) [26.0-57.0]	NA	NA
Missing, No.	29	21	NA	NA
Parenting stress score				
Mean (SD) [range]	111.8 (21.0) [66.0-181.0]	115.2 (22.9) [66.0-187.0]	NA	NA
Missing, No.	27	20	NA	NA
Child age, mean (SD) [range], y	1.6 (0.1) [1.2-2.2]	3.2 (0.2) [2.8-4.0]	4.9 (0.2) [4.4-5.5]	8.4 (0.5) [7.9-11.9]
Internalizing behaviors score				
Mean (SD) [range]	44.9 (9.3) [29.0-83.0]	48.0 (9.9) [29.0-78.0]	49.0 (12.0) [29.0-78.0]	54.1 (10.1) [33.0-82.0]
Clinical problems, No.	3	11	20	24
Missing, No.	27	17	24	33

### Hypothesis 1: Trajectories of Internalizing Behaviors

In Table 3 the final multilevel models are provided. The data were suited to multilevel analysis, with 44% of the variance in CBCL-Internalizing behaviors attributed to interchild differences. At 1.5 years (intercept), internalizing behavior scores were within normal range (mean [SD] T-score, 45.47 [7.50]). On average, as child age increased, CBCL-Internalizing scores were higher (Table 3, model 1), with the model estimates showing an increase of 8.5 T-score points across ages 1.5 through 8.0 years. Next, gestational age at birth was added in model 2. Higher internalizing behaviors were associated with lower gestational age at birth (a T-score increase of 0.6 per week born earlier) (Table 3, model 2). Above and beyond clinical factors related to prematurity and gestational age at birth, greater exposure to neonatal pain-related stress was associated with increased internalizing behaviors across age (B = 4.95; 95% CI, 0.76 to 9.14) (Table 3, model 3). Neonatal pain-related stress did not vary by child age at assessment in relation to internalizing behaviors.

**Table 3.** Final Models of Factors Associated With Increased Child Internalizing Behaviors Across Ages 1.5, 3.0, 4.5, and 8.0 Years

Study variable	B (95% CI)			
	Model 1	Model 2	Model 3	Model 4
Child age at visit	1.29 (1.05 to 1.52)	1.29 (1.06 to 1.52)	1.29 (1.06 to 1.52)	1.31 (1.07 to 1.54)
Gestational age at birth	NA	−0.63 (−1.17 to −0.08)	−0.39 (−1.42 to 0.64)	−1.08 (−2.08 to −0.07)
Child sex^a^	NA	NA	2.03 (−0.42 to 4.49)	1.56 (−0.79 to 3.91)
Illness severity (day 1)	NA	NA	0.09 (−0.01 to 0.19)	0.11 (0.02 to 0.21)
Neonatal pain-related stress	NA	NA	4.95 (0.76 to 9.14)	2.92 (−1.09 to 6.94)
Cumulative morphine exposure	NA	NA	−0.73 (−5.11 to 3.66)	−2.98 (−7.79 to 1.84)
Days receiving mechanical ventilation and/or oscillation	NA	NA	−0.69 (−1.54 to 0.16)	−0.96 (−1.98 to 0.05)
Culture-positive infection^b^	NA	NA	−1.42 (−4.74 to 1.90)	−1.50 (−4.73 to 1.72)
No. of surgical procedures	NA	NA	0.19 (−1.55 to 1.94)	0.39 (−1.27 to 2.04)
Parenting stress at 1.5 y	NA	NA	NA	0.17 (0.11 to 0.23)
Parent environment at 1.5 y^c^				
Low support	NA	NA	NA	0.74 (−2.79 to 4.26)
High support	NA	NA	NA	1.31 (−3.73 to 6.35)
Child interactive behavior at 1.5 y	NA	NA	NA	−0.29 (−0.56 to −0.04)

^a^ Male = 0.

^b^ Confirmed infection = 1.

^c^ Reference group was parent environment 1.5 years: average support.

### Hypothesis 2: Profiles of Parent Environment

#### Age 1.5 Years

**Figure 1.**
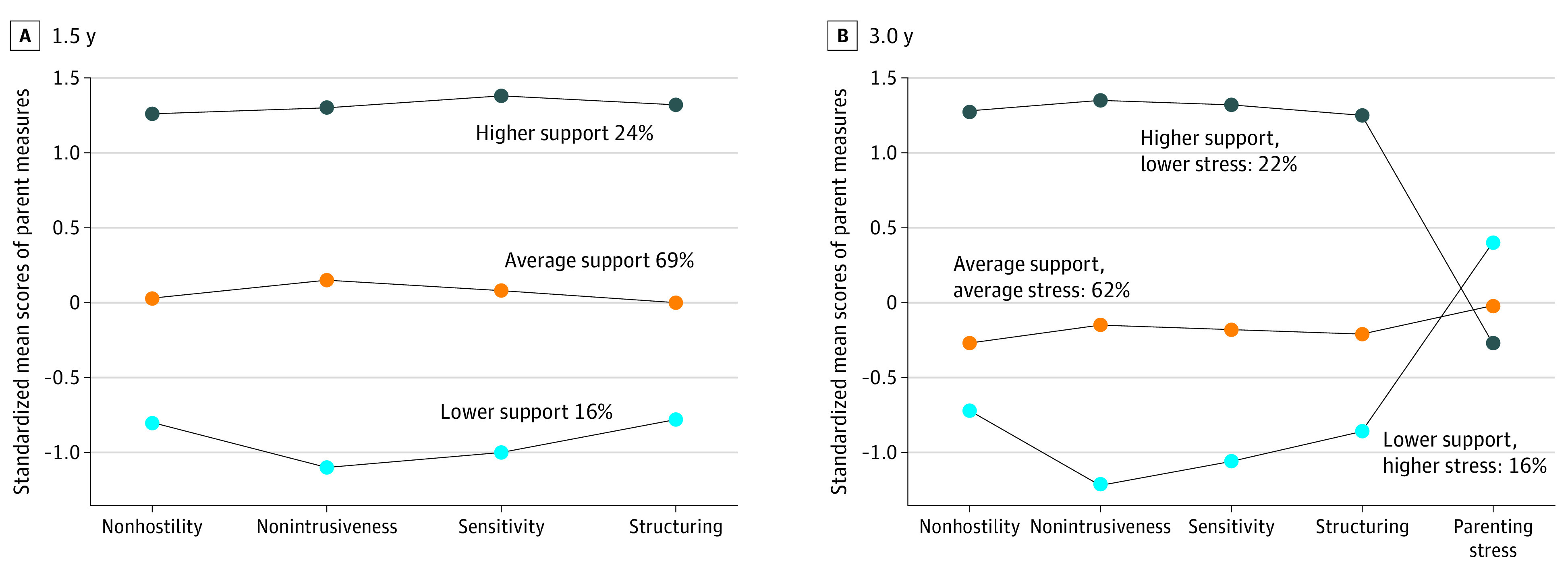
Profiles of Parent Environment Scores are mean standardized scores at 1.5 years considering only parent behaviors (A) and at 3.0 years considering parenting stress and parent behaviors (B). The scales across Parenting Stress Index and Emotional Availability Scale–IV measures vary.

At this age, when considering parenting stress and parent behaviors together in latent profile analyses, statistically, the bayesian information criterion (BIC) index identified 1 profile of parent environment. However, the uncertainty plots of classification and bootstrapped sequential LRTs indicated considerable variability in parent behaviors and parenting stress at this age (Table 2). Therefore, we only included parenting behaviors in subsequent latent profile analysis and considered parenting stress as a separate factor in models. The 3 profiles are displayed in Figure 1A (higher support, 24%; average support, 69%; lower support, 16%). Although a 2-factor model demonstrated slightly better fit (BIC, −1203.8; integrated complete-data likelihood [ICL], −1221.8) than the 3-factor model (BIC, −1219.0; ICL, −1268.0), inspection of uncertainty of classification plots revealed better model convergence with the 3-factor model. Bootstrapped sequential LRTs confirmed suitability of the 3-factor vs 2-factor model (LRT, 43.35; *P* = .001). Moreover, the 3 profiles better differentiated between established EA Parent Dimension subscales and, thus, theoretical models of emotional availability.^25^ Parents demonstrating low and average support behavior profiles displayed inconsistent EA, with profiles differing primarily in their levels of nonintrusiveness, with low support parents demonstrating nonoptimal behaviors (interfering, directing, and driving play). In subsequent modeling, we tested both the additive and interactive effects of our profiles (herein termed “parent support”) and parenting stress at 1.5 years in association with internalizing behaviors.

#### Age 3.0 Years

Using the BIC index for model fit, the best fitting model (BIC, −1568.0; ICL, 1586.0) identified a 2-factor model for parent environment at age 3.0 years. Similar to our findings at age 1.5 years, inspection of uncertainty of classification plots revealed better model convergence with the 3-factor than 2-factor model. Bootstrapped LRTs further confirmed suitability of the 3-factor vs 2-factor model (LRT, 43.36; *P* = .001). The 3 parenting profiles identified at age 3.0 years are presented in Figure 1B (high supportive parenting behaviors, low parenting stress, 22%; average supportive parenting behaviors and average parenting stress levels, 62%; low supportive parenting behaviors and high parenting stress, 16%). Parents in the high support, low stress profile displayed optimal parenting across all behaviors (EA scores of approximately 26), and typical stress (24th percentile). In contrast, parents who displayed a low support, average high stress profile displayed, on average, nonoptimal parenting behaviors and elevated, albeit typical stress (50th percentile). Similar to profiles at 1.5 years, parent nonintrusiveness played a key role in construction of the low support, high stress parent behavior profile.

### Hypothesis 3

#### Parenting at 1.5 Years

Higher parenting stress at age 1.5 years (B = 0.17; 95% CI, 0.11 to 0.23), but not parent support, was associated with higher internalizing behaviors across childhood (Table 3, model 4). After consideration of parenting stress at 1.5 years, neonatal pain-related stress was no longer significantly associated with internalizing behaviors. Profiles of parent support and parenting stress did not interact in association with internalizing behaviors (*P* for interaction = .08). Model 4 accounted for 51% of variance in child internalizing behaviors (21% within-participant differences including child age; 30% between-participant variables). Parenting stress and parent support at 1.5 years did not differ by neonatal pain-related stress or gestational age at birth across child age at assessment in association with internalizing behaviors.

#### Parenting at 3.0 Years

**Figure 2.**
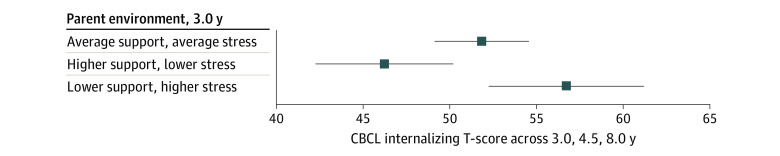
Parent Environment at Age 3.0 Years Associated With Internalizing Behaviors Across Ages 3.0, 4.5, and 8.0 Years Error bars denote 95% CIs. The model (model 4) is reported in eTable 4 in the Supplement. CBCL indicates Child Behavior Checklist.

To examine associations of parenting at 3.0 years with internalizing behaviors and neonatal factors associated with prematurity, models were rerun considering only internalizing behaviors from 3.0 through 8.0 years (eTable 4 in the Supplement). Parent environment at 3.0 years was significantly associated with trajectories of internalizing behaviors, such that child internalizing behaviors were highest for those children exposed to a low interaction support, high stress parent environment at age 3.0 years (higher support, lower stress, B = −5.47; 95% CI, −9.44 to −1.51) (Figure 2 and eTable 4, model 4 in the Supplement). After considering profiles of parent environment at 3.0 years, neonatal pain-related stress was no longer significantly associated with child internalizing behaviors. The final model accounted for 59% of the variance in child internalizing behaviors (44% attributable to between-individual differences in internalizing at 3.0 years, 15% accounted for by model variables). Importantly, the association between parent environment and internalizing behaviors across development held after accounting for child interactive behaviors during semistructured play and maternal education (eTable 5 [model 5] and eTable 6 [model 4] in the Supplement).

Associations of parent environment at 3.0 years with internalizing behaviors across ages were not moderated by neonatal pain-related stress or gestational age at birth across child age at assessment. The final model considering parenting behaviors at 3.0 years held when rerun considering internalizing behaviors across 1.5, 3.0, 4.5, and 8.0 years (eTable 5 [model 5] in the Supplement). Model estimates were comparable when using complete data (eTable 5 and eTable 6 in the Supplement).

## Discussion

In this prospective, longitudinal cohort study, we identified environmental factors across the first 3 years of life associated with trajectories of early internalizing behaviors in children born very preterm. We found that parent-reported internalizing behaviors increased from 1.5, 3.0, 4.5, to 8.0 years. First, we showed that, above and beyond gestational age at birth and neonatal clinical factors, greater pain-related stress was associated with greater internalizing behaviors across this period. In subsequent models in which we considered the more proximal parent environment, neonatal pain-related stress was no longer associated with outcomes. Instead, elevated internalizing behaviors were associated with greater parenting stress at 1.5 years, whereas a more supportive parent environment at age 3.0 years was associated with fewer internalizing behaviors across 1.5 through 8.0 years. Our findings have implications for follow-up monitoring of children born very preterm and delivery of interventions in pediatric care.

Our finding of increasing internalizing behavior symptoms across 1.5, 3.0, 4.5, and 8.0 years is in line with prior work examining trajectories through childhood^38^ to adolescence^1,3^ in children born very preterm. In the current study, the majority of children displayed parent-reported internalizing behaviors within the normative range at 1.5 years; however, by 8.0 years, almost 1 in 7 children displayed behaviors in the clinically abnormal range. Internalizing problems are related to poorer school^39,40^ and social^41^ functioning and, later, psychiatric illness.^1^ Our findings support the continued monitoring of children born very preterm through early childhood.

When considering only the early NICU environment, we found that exposure to neonatal pain-related stress was associated with higher internalizing symptoms across 1.5, 3.0, 4.5, and 8.0 years, after accounting for clinical risk factors. This association was stable across early childhood. These findings extend our prior work in an independent cohort in which we demonstrated that NICU pain-related stress is associated with greater internalizing behaviors at ages 1.5 years^9^ and 8.0 years.^18^ Alterations to brain development in key stress-related limbic regions as early as the neonatal period^42,43^ and evident at school age^44^ may underlie poorer behavioral outcomes independently of, or in interaction with, stress dysregulation (hypothalamic pituitary adrenal axis),^7,45,46,47^ which is also related to internalizing behaviors.^7,45^ Across cohorts of children born very preterm, we have demonstrated that exposure to neonatal pain-related stress is associated with widespread difficulties in neurobehavioral outcomes, including cognition, motor skills, and behavior across childhood.^15^

Our most novel and important finding in the present study is that a supportive parent environment reflecting more positive parent behaviors and lower stress was associated with a reduction in the development of internalizing behaviors across early childhood in children born very preterm. These findings are in concert with prior work^9,20,21,22,48,49^ largely limited to cross-sectional study designs investigating specific parent behaviors without considering the related influence of parenting stress. Extending on evidence from an experimental animal model of neonatal pain^50^ and our prior work,^9,45,51^ here we show that a positive postnatal environment, inclusive of supportive, structured maternal behaviors and mood may attenuate, whereas negative environments may maintain, adverse outcomes associated with neonatal pain-related stress exposure and other factors associated with prematurity.

Beyond effects of preterm birth, children exposed to more neonatal pain-related stress display behavioral dysregulation, poorer physiological stress regulation, sensory processing problems,^52^ and early attentional problems associated with poorer executive functioning.^15^ Sensitive, warm, and nonintrusive parents who provide structured guidance may attenuate the development of children’s internalizing behavior by helping their child cope with feelings of anxiety and the tendency to withdraw in threatening situations,^53,54^ promote child attention and engagement in tasks,^55^ support healthy physiological stress regulation,^7^ and promote behavioral regulatory skills. However, poor parent well-being and parenting stress, which are common in parents of preterm infants,^56^ make providing a supportive environment challenging for parents^51^ and impede appropriate modeling of anxiety-related behaviors.^57,58^ Of note, disentangling the directionality of parent-child associations is difficult. Poorer neurodevelopment^37,38,39^ and difficult temperament^59^ in preterm born children are associated with persistent parenting stress^59,60^ and are challenging for parenting.^61^ Although there is evidence that parents experience more stress when their child is challenged developmentally,^62^ other research suggests that parent contribution is independent of child developmental challenge.^21,63^ Our findings more likely support the latter, as the association between parenting and child internalizing behaviors across development was evident after accounting for child behavior when assessing parent behavior during parent-child semistructured teaching interaction.

At 3.0 years, but not 1.5 years, children displayed lower parent-reported internalizing behaviors related to supportive parenting interactions, after accounting for observed child behavior during parent-child semistructured teaching task. Although prior work demonstrates that positive parenting in the NICU through the first 3 years has immediate benefits,^9^ few have examined the sustained benefits of early parenting behaviors as they align with developmental timing.^21,64^ We postulate that parenting behaviors in the context of teaching tasks may be most beneficial for long-term behavioral outcomes in late toddlerhood, when children shift from emotional coregulation strategies dependent on their primary caregiver toward activities of exploration and independent learning.^65^ Around age 3.0 years, self-regulatory skills^66^ and related executive functioning (eg, attentional shifting and inhibition) abilities are emerging.^55^ At this age, preterm born children may benefit from structured guidance that encourages autonomy. Our findings have implications for integration of parent support intervention in neonatal follow-up. Parent-led interventions that target parent psychosocial support and supportive training in multiple developmentally appropriate behaviors may prove beneficial. Importantly, such interventions may help prepare parents and children for the evolving developmental needs and environmental changes into the school years.^67^

Significant participant retention across all 4 developmental visits is a clear strength of our study. By excluding children with severe brain injury and/or major impairments, our findings represent outcomes for the broad majority of children born very preterm. Modeling accounted for clinical confounders in an attempt to disentangle the role of pain and/or stress from the reasons why procedures are performed (eg, infection). Future research could look to examine other between-participant factors related to the onset and development of internalizing behaviors in this population, including potential genetic susceptibility.

### Limitations

Our study has several limitations. The parents in our cohort had high education levels, with universal access to health care. Importantly, despite this, the associations between parenting and child internalizing behaviors remained after accounting for parent education. We did not collect data on individual early allied health interventions, access to which varies by region in British Columbia. We examined parenting in the context of semistructured teaching tasks only; however, given the high need for structuring for children born very preterm, we chose this a key context for parent factors to be examined. The lack of parent-child interaction and parenting stress data at ages 4.5 and 8.0 years did not allow for examination of potential bidirectional parent-child relationships across development; this is an important question for future research.

## Conclusions

In a prospective cohort study of children born very preterm, we demonstrate associations among aspects of the early environment and development of internalizing behaviors across childhood. We found that neonatal pain-related stress is related to the early emergence of these behaviors, and parents play a pivotal role in attenuating child behavior problems across development. Importantly, our novel findings highlight parenting as a modifiable factor related to better outcomes for this vulnerable population, not only in the short term but with continued benefits as children develop. Our findings warrant support of parents of children born very preterm across the early years and development and implementation of efficacious parent-child intervention strategies that promote optimal behavior in this population.

## Notes

Supplement. **eFigure 1.** Cohort Flowchart **eAppendix 1.** Supplemental Methods **eAppendix 2.** Statistical Analysis **eFigure 2.** Patterns of Missing Data **eTable 1.** Missing Data for Each Study Variable **eAppendix 3.** Supplemental Results **eTable 2.** Intercorrelations Among CBCL Internalizing T-Scores, Parent and Child During Semistructured Teaching Task Within and Across Ages 1.5 and 3 Years **eTable 3.** Demographic Comparison of Children With Complete Versus Incomplete Data **eTable 4.** Final Models of Factors Related to Increased Child Internalizing Behaviors Across 3, 4.5, 8 Years Using Imputed Data **eTable 5.** Estimated Multilevel Models of Internalizing Behaviors Across 1.5, 3, 4.5, 8 Years Using Listwise Deletion for Missing Data **eTable 6.** Estimated Multilevel Models of Internalizing Behaviors Across 3, 4.5, 8 Years Using Listwise Deletion for Missing Data
eReferences

## References

[1] Johnson S, Marlow N. Preterm birth and childhood psychiatric disorders. Pediatr Res. 2011;69(5 pt 2):11R-18R. doi:10.1203/PDR.0b013e318212faa0 21289534

[2] Johnson S. Cognitive and behavioural outcomes following very preterm birth. Semin Fetal Neonatal Med. 2007;12(5):363-373. doi:10.1016/j.siny.2007.05.004 17625996

[3] Linsell L, Johnson S, Wolke D, Morris J, Kurinczuk JJ, Marlow N. Trajectories of behavior, attention, social and emotional problems from childhood to early adulthood following extremely preterm birth: a prospective cohort study. Eur Child Adolesc Psychiatry. 2019;28(4):531-542. doi:10.1007/s00787-018-1219-8 30191335 PMC6445809

[4] Burnett AC, Anderson PJ, Cheong J, Doyle LW, Davey CG, Wood SJ. Prevalence of psychiatric diagnoses in preterm and full-term children, adolescents and young adults: a meta-analysis. Psychol Med. 2011;41(12):2463-2474. doi:10.1017/S003329171100081X 21733218

[5] Grunau RE, Whitfield MF, Fay TB. Psychosocial and academic characteristics of extremely low birth weight (≤800 g) adolescents who are free of major impairment compared with term-born control subjects. Pediatrics. 2004;114(6):e725-e732. doi:10.1542/peds.2004-0932 15576337

[6] Gerstein ED, Woodman AC, Burnson C, Cheng ER, Poehlmann-Tynan J. Trajectories of externalizing and internalizing behaviors in preterm children admitted to a neonatal intensive care unit. J Pediatr. 2017;187:111-118. doi:10.1016/j.jpeds.2017.04.047 28533035 PMC5533642

[7] Brummelte S, Grunau RE, Zaidman-Zait A, Weinberg J, Nordstokke D, Cepeda IL. Cortisol levels in relation to maternal interaction and child internalizing behavior in preterm and full-term children at 18 months corrected age. Dev Psychobiol. 2011;53(2):184-195. doi:10.1002/dev.20511 21298633 PMC3099144

[8] Guilherme Monte Cassiano R, Gaspardo CM, Cordaro Bucker Furini G, Martinez FE, Martins Linhares MB. Impact of neonatal risk and temperament on behavioral problems in toddlers born preterm. Early Hum Dev. 2016;103:175-181. doi:10.1016/j.earlhumdev.2016.09.015 27701040

[9] Vinall J, Miller SP, Synnes AR, Grunau RE. Parent behaviors moderate the relationship between neonatal pain and internalizing behaviors at 18 months corrected age in children born very prematurely. Pain. 2013;154(9):1831-1839. doi:10.1016/j.pain.2013.05.050 23748079 PMC3791108

[10] Scott A, Winchester SB, Sullivan MC. Trajectories of problem behaviors from 4 to 23 years in former preterm infants. Int J Behav Dev. 2018;42(2):237-247. doi:10.1177/0165025417692899 29430071 PMC5805147

[11] Yates R, Treyvaud K, Doyle LW, . Rates and stability of mental health disorders in children born very preterm at 7 and 13 years. Pediatrics. 2020;145(5):e20192699. doi:10.1542/peds.2019-2699 32276969

[12] Institute of Medicine (US) Committee on Understanding Premature Birth and Assuring Healthy Outcomes. In: Behrman RE, Butler AS, eds. Preterm Birth: Causes, Consequences, and Prevention. National Academies Press; 2007. Accessed August 19, 2021. https://www.ncbi.nlm.nih.gov/books/NBK11362/20669423

[13] Carbajal R, Rousset A, Danan C, . Epidemiology and treatment of painful procedures in neonates in intensive care units. JAMA. 2008;300(1):60-70. doi:10.1001/jama.300.1.60 18594041

[14] Hatfield LA. Neonatal pain: what’s age got to do with it? Surg Neurol Int. 2014;5(14)(suppl 13):S479-S489. doi:10.4103/2152-7806.144630 25506507 PMC4253046

[15] Grunau RE, Vinall Miller J, Chau CMY. Long-term effects of pain in infants. In: Stevens BJ, Hathway G, Zempsky WT, eds. Oxford Textbook of Pediatric Pain. Oxford University Press; 2021:38-46. doi:10.1093/med/9780198818762.003.0004

[16] Fitzgerald M. The development of central nociceptive processing and descending modulation of pain. In: Stevens BJ, Hathway G, Zempsky WT, eds. Oxford Textbook of Pediatric Pain. Oxford University Press; 2021:72-78. doi:10.1093/med/9780198818762.003.0008

[17] Moriarty O, Walker SM. Long-term effects of early pain and injury: Animal models. In: Stevens BJ, Hathway G, Zempsky WT, eds. Oxford Textbook of Pediatric Pain. Oxford University Press; 2021:21-37. doi:10.1093/med/9780198818762.003.0003

[18] Ranger M, Synnes AR, Vinall J, Grunau RE. Internalizing behaviours in school-age children born very preterm are predicted by neonatal pain and morphine exposure. Eur J Pain. 2014;18(6):844-852. doi:10.1002/j.1532-2149.2013.00431.x 24318537 PMC4016156

[19] Treyvaud K, Anderson VA, Howard K, . Parenting behavior is associated with the early neurobehavioral development of very preterm children. Pediatrics. 2009;123(2):555-561. doi:10.1542/peds.2008-0477 19171622

[20] Neel MLM, Stark AR, Maitre NL. Parenting style impacts cognitive and behavioural outcomes of former preterm infants: a systematic review. Child Care Health Dev. 2018;44(4):507-515. doi:10.1111/cch.12561 29575031 PMC6005730

[21] Treyvaud K, Doyle LW, Lee KJ, . Parenting behavior at 2 years predicts school-age performance at 7 years in very preterm children. J Child Psychol Psychiatry. 2016;57(7):814-821. doi:10.1111/jcpp.12489 26616792

[22] Stack DM, Matte-Gagné C, Dickson DJ. Persistence of effects of VLBW/PT birth status and maternal emotional availability (EA) on child EA trajectories. Front Psychol. 2019;9:2715. doi:10.3389/fpsyg.2018.02715 30761058 PMC6361804

[23] Grunau RE, Whitfield MF, Petrie-Thomas J, . Neonatal pain, parenting stress and interaction, in relation to cognitive and motor development at 8 and 18 months in preterm infants. Pain. 2009;143(1-2):138-146. doi:10.1016/j.pain.2009.02.014 19307058 PMC2836793

[24] Abidin RR. Parenting Stress Index. Psychological Assessment Resources; 1995.

[25] Biringen Z. Emotional availability: conceptualization and research findings. Am J Orthopsychiatry. 2000;70(1):104-114. doi:10.1037/h0087711 10702855

[26] Clark ELM, Jiao Y, Sandoval K, Biringen Z. Neurobiological implications of parent-child emotional availability: a review. Brain Sci. 2021;11(8):1016. doi:10.3390/brainsci11081016 34439635 PMC8391119

[27] Saunders H, Kraus A, Barone L, Biringen Z. Emotional availability: theory, research, and intervention. Front Psychol. 2015;6:1069. doi:10.3389/fpsyg.2015.01069 26283996 PMC4516809

[28] Achenbach TM, Rescorla L. Manual for the ASEBA Preschool Form & Profiles. University of Vermont, Research Center for Children, Youth and Families; 2000.

[29] Achenbach TM, Rescorla L. Manual for the ASEBA School-Age Forms & Profiles. University of Vermont, Research Centre for Children, Youth, & Families; 2001.

[30] R Core Team. R: a language and environment for statistical computing. 2021. Accessed September 15, 2022. https://www.R-project.org/

[31] Scrucca L, Fop M, Murphy TB, Raftery AE. mclust 5: Clustering, classification and density estimation using Gaussian finite mixture models. R J. 2016;8(1):289-317. doi:10.32614/RJ-2016-021 27818791 PMC5096736

[32] Bates D, Mächler M, Bolker B, Walker S. Fitting linear mixed-effects models using lme4. J Stat Softw. 2015;67(1):1-48. doi:10.18637/jss.v067.i01

[33] Kuznetsova A, Brockhoff PB, Christensen RHB. lmerTest package: tests in linear mixed effects models. J Stat Softw. 2017;82(13). doi:10.18637/jss.v082.i13

[34] Carpenter JR, Kenward MG. Multiple Imputation and Its Application. John Wiley & Sons, Ltd; 2013. doi:10.1002/9781119942283

[35] Quartagno M, Carpenter J. Jomo: a package for multilevel joint modelling multiple imputation. 2020. Accessed September 15, 2022. https://CRAN.R-project.org/package=jomo

[36] Grund S, Robitzsch A, Luedtke O. Mitml: tools for multiple imputation in multilevel modeling. 2021. Accessed September 15, 2022. https://CRAN.R-project.org/package=mitml

[37] Meng XL, Rubin DB. Performing likelihood ratio tests with multiply-imputed data sets. Biometrika. 1992;79(1):103-111. doi:10.1093/biomet/79.1.103

[38] Arpi E, Ferrari F. Preterm birth and behaviour problems in infants and preschool-age children: a review of the recent literature. Dev Med Child Neurol. 2013;55(9):788-796. doi:10.1111/dmcn.12142 23521214

[39] Mychailyszyn MP, Kendall PC, Mendez JL. School functioning in youth with and without anxiety disorders: comparisons by diagnosis and comorbidity. School Psych Rev. 2010;39(1):106-121. doi:10.1080/02796015.2010.12087793

[40] Breslau J, Lane M, Sampson N, Kessler RC. Mental disorders and subsequent educational attainment in a US national sample. J Psychiatr Res. 2008;42(9):708-716. doi:10.1016/j.jpsychires.2008.01.016 18331741 PMC2748981

[41] Settipani CA, Kendall PC. Social functioning in youth with anxiety disorders: association with anxiety severity and outcomes from cognitive-behavioral therapy. Child Psychiatry Hum Dev. 2013;44(1):1-18. doi:10.1007/s10578-012-0307-0 22581270

[42] Brummelte S, Grunau RE, Chau V, . Procedural pain and brain development in premature newborns. Ann Neurol. 2012;71(3):385-396. doi:10.1002/ana.22267 22374882 PMC3760843

[43] Duerden EG, Grunau RE, Guo T, . Early procedural pain is associated with regionally-specific alterations in thalamic development in preterm neonates. J Neurosci. 2018;38(4):878-886. doi:10.1523/JNEUROSCI.0867-17.2017 29255007 PMC5783966

[44] Chau CMY, Ranger M, Bichin M, . Hippocampus, amygdala, and thalamus volumes in very preterm children at 8 years: neonatal pain and genetic variation. Front Behav Neurosci. 2019;13:51. doi:10.3389/fnbeh.2019.00051 30941021 PMC6433974

[45] Brummelte S, Chau CMY, Cepeda IL, . Cortisol levels in former preterm children at school age are predicted by neonatal procedural pain-related stress. Psychoneuroendocrinology. 2015;51:151-163. doi:10.1016/j.psyneuen.2014.09.018 25313535 PMC4268136

[46] Grunau RE, Weinberg J, Whitfield MF. Neonatal procedural pain and preterm infant cortisol response to novelty at 8 months. Pediatrics. 2004;114(1):e77-e84. doi:10.1542/peds.114.1.e77 15231977 PMC1351380

[47] Grunau RE, Cepeda IL, Chau CMY, . Neonatal pain-related stress and *NFKBIA* genotype are associated with altered cortisol levels in preterm boys at school age. PLoS One. 2013;8(9):e73926. doi:10.1371/journal.pone.0073926 24066085 PMC3774765

[48] Windhorst DA, Rippe RCA, Mileva-Seitz VR, . Mild perinatal adversities moderate the association between maternal harsh parenting and hair cortisol: evidence for differential susceptibility. Dev Psychobiol. 2017;59(3):324-337. doi:10.1002/dev.21497 28295227

[49] Poehlmann J, Hane A, Burnson C, Maleck S, Hamburger E, Shah PE. Preterm infants who are prone to distress: differential effects of parenting on 36-month behavioral and cognitive outcomes. J Child Psychol Psychiatry. 2012;53(10):1018-1025. doi:10.1111/j.1469-7610.2012.02564.x 22582942 PMC3467011

[50] Mooney-Leber SM, Brummelte S. Neonatal pain and reduced maternal care alter adult behavior and hypothalamic-pituitary-adrenal axis reactivity in a sex-specific manner. Dev Psychobiol. 2020;62(5):631-643. doi:10.1002/dev.21941 31788799

[51] Tu MT, Grunau RE, Petrie-Thomas J, Haley DW, Weinberg J, Whitfield MF. Maternal stress and behavior modulate relationships between neonatal stress, attention, and basal cortisol at 8 months in preterm infants. Dev Psychobiol. 2007;49(2):150-164. doi:10.1002/dev.20204 17299787 PMC1851900

[52] McLean MA, Niknafs N, Scoten OC, . Sensory processing and cortisol at age 4 years: procedural pain-related stress in children born very preterm. Dev Psychobiol. 2021;63(5):915-930. doi:10.1002/dev.22079 33377181

[53] Kok R, Linting M, Bakermans-Kranenburg MJ, . Maternal sensitivity and internalizing problems: evidence from two longitudinal studies in early childhood. Child Psychiatry Hum Dev. 2013;44(6):751-765. doi:10.1007/s10578-013-0369-7 23408268

[54] van der Voort A, Linting M, Juffer F, Bakermans-Kranenburg MJ, Schoenmaker C, van Ijzendoorn MH. The development of adolescents’ internalizing behavior: longitudinal effects of maternal sensitivity and child inhibition. J Youth Adolesc. 2014;43(4):528-540. doi:10.1007/s10964-013-9976-7 23828726

[55] Valcan DS, Davis H, Pino-Pasternak D. Parental behaviours predicting early childhood executive functions: a meta-analysis. Educ Psychol Rev. 2018;30(3):607-649. doi:10.1007/s10648-017-9411-9

[56] Trumello C, Candelori C, Cofini M, . Mothers’ depression, anxiety, and mental representations after preterm birth: a study during the infant’s hospitalization in a neonatal intensive care unit. Front Public Health. 2018;6:359. doi:10.3389/fpubh.2018.00359 30581812 PMC6293875

[57] Cobham VE, Dadds MR, Spence SH. The role of parental anxiety in the treatment of childhood anxiety. J Consult Clin Psychol. 1998;66(6):893-905. doi:10.1037/0022-006X.66.6.893 9874902

[58] Hudson JL, Dodd HF, Bovopoulos N. Temperament, family environment and anxiety in preschool children. J Abnorm Child Psychol. 2011;39(7):939-951. doi:10.1007/s10802-011-9502-x 21479669

[59] Gray PH, Edwards DM, O’Callaghan MJ, Cuskelly M, Gibbons K. Parenting stress in mothers of very preterm infants: influence of development, temperament and maternal depression. Early Hum Dev. 2013;89(9):625-629. doi:10.1016/j.earlhumdev.2013.04.005 23669559

[60] Gray PH, Edwards DM, Gibbons K. Parenting stress trajectories in mothers of very preterm infants to 2 years. Arch Dis Child Fetal Neonatal Ed. 2018;103(1):F43-F48. doi:10.1136/archdischild-2016-312141 28659361

[61] Salvatori P, Neri E, Chirico I, Andrei F, Agostini F, Trombini E. Mother-toddler play interaction in extremely, very low birth weight, and full-term children: a longitudinal study. Front Psychol. 2016;7:1511. doi:10.3389/fpsyg.2016.01511 27746756 PMC5043650

[62] Brummelte S, Grunau RE, Synnes AR, Whitfield MF, Petrie-Thomas J. Declining cognitive development from 8 to 18 months in preterm children predicts persisting higher parenting stress. Early Hum Dev. 2011;87(4):273-280. doi:10.1016/j.earlhumdev.2011.01.030 21334150

[63] Landry SH, Smith KE, Swank PR, Miller-Loncar CL. Early maternal and child influences on children’s later independent cognitive and social functioning. Child Dev. 2000;71(2):358-375. doi:10.1111/1467-8624.00150 10834470

[64] Faure N, Habersaat S, Harari MM, . Maternal sensitivity: a resilience factor against internalizing symptoms in early adolescents born very preterm? J Abnorm Child Psychol. 2017;45(4):671-680. doi:10.1007/s10802-016-0194-0 27573689

[65] Humphreys KL, Zeanah CH, Scheeringa MS. Infant development: the first 3 years of life. In: Tasman A, Kay J, Lieberman JA, First MB, Riba MB, eds. Psychiatry, 4th ed. John Wiley & Sons, Ltd; 2015:134-158. doi:10.1002/9781118753378.ch9

[66] Kopp CB. Antecedents of self-regulation: a developmental perspective. Dev Psychol. 1982;18(2):199-214. doi:10.1037/0012-1649.18.2.199

[67] Anderson PJ, Treyvaud K, Spittle AJ. Early developmental interventions for infants born very preterm: what works? Semin Fetal Neonatal Med. 2020;25(3):101119. doi:10.1016/j.siny.2020.101119 32446767

