# Supplementary material for: Association of Neonatal Pain-Related Stress and Parent Interaction With Internalizing Behaviors Across 1.5, 3.0, 4.5, and 8.0 Years in Children Born Very Preterm
Source: JAMA Netw Open. 2022 Oct 21;5(10):e2238088. doi: 10.1001/jamanetworkopen.2022.38088 (PMC9587482; doi:10.1001/jamanetworkopen.2022.38088)
Supplement: Supplement. — eFigure 1. Cohort Flowchart eAppendix 1. Supplemental Methods eAppendix 2. Statistical Analysis eFigure 2. Patterns of Missing Data eTable 1. Missing Data for Each Study Variable eAppendix 3. Supplemental Results eTable 2. Intercorrelations Among CBCL Internalizing T-Scores, Parent and Child During Semistructured Teaching Task Within and Across Ages 1.5 and 3 Years eTable 3. Demographic Comparison of Children With Complete Versus Incomplete Data eTable 4. Final Models of Factors Related to Increased Child Internalizing Behaviors Across 3, 4.5, 8 Years Using Imputed Data eTable 5. Estimated Multilevel Models of Internalizing Behaviors Across 1.5, 3, 4.5, 8 Years Using Listwise Deletion for Missing Data eTable 6. Estimated Multilevel Models of Internalizing Behaviors Across 3, 4.5, 8 Years Using Listwise Deletion for Missing Data eReferences [file jamanetwopen-e2238088-s001.pdf]

Supplemental Online Content

McLean MA, Scoten OC, Chau CMY, Synnes A, Miller SP, Grunau RE. Association of neonatal pain-related stress and parent interaction with internalizing behaviors across 1.5, 3.0, 4.5, and 8.0 years in children born very preterm. *JAMA Netw Open*. 2022;5(10):e2238088. doi:10.1001/jamanetworkopen.2022.38088

eFigure 1. Cohort Flowchart

eAppendix 1. Supplemental Methods

eAppendix 2. Statistical Analysis

eFigure 2. Patterns of Missing Data

eTable 1. Missing Data for Each Study Variable

eAppendix 3. Supplemental Results

eTable 2. Intercorrelations Among CBCL Internalizing T-Scores, Parent and Child During Semistructured Teaching Task Within and Across Ages 1.5 and 3 Years

eTable 3. Demographic Comparison of Children With Complete Versus Incomplete Data

eTable 4. Final Models of Factors Related to Increased Child Internalizing Behaviors Across 3, 4.5, 8 Years Using Imputed Data

eTable 5. Estimated Multilevel Models of Internalizing Behaviors Across 1.5, 3, 4.5, 8 Years Using Listwise Deletion for Missing Data

eTable 6. Estimated Multilevel Models of Internalizing Behaviors Across 3, 4.5, 8 Years Using Listwise Deletion for Missing Data

eReferences

This supplemental material has been provided by the authors to give readers additional information about their work.

© 2022 McLean MA et al. *JAMA Network Open.*

eFigure 1. Cohort Flowchart

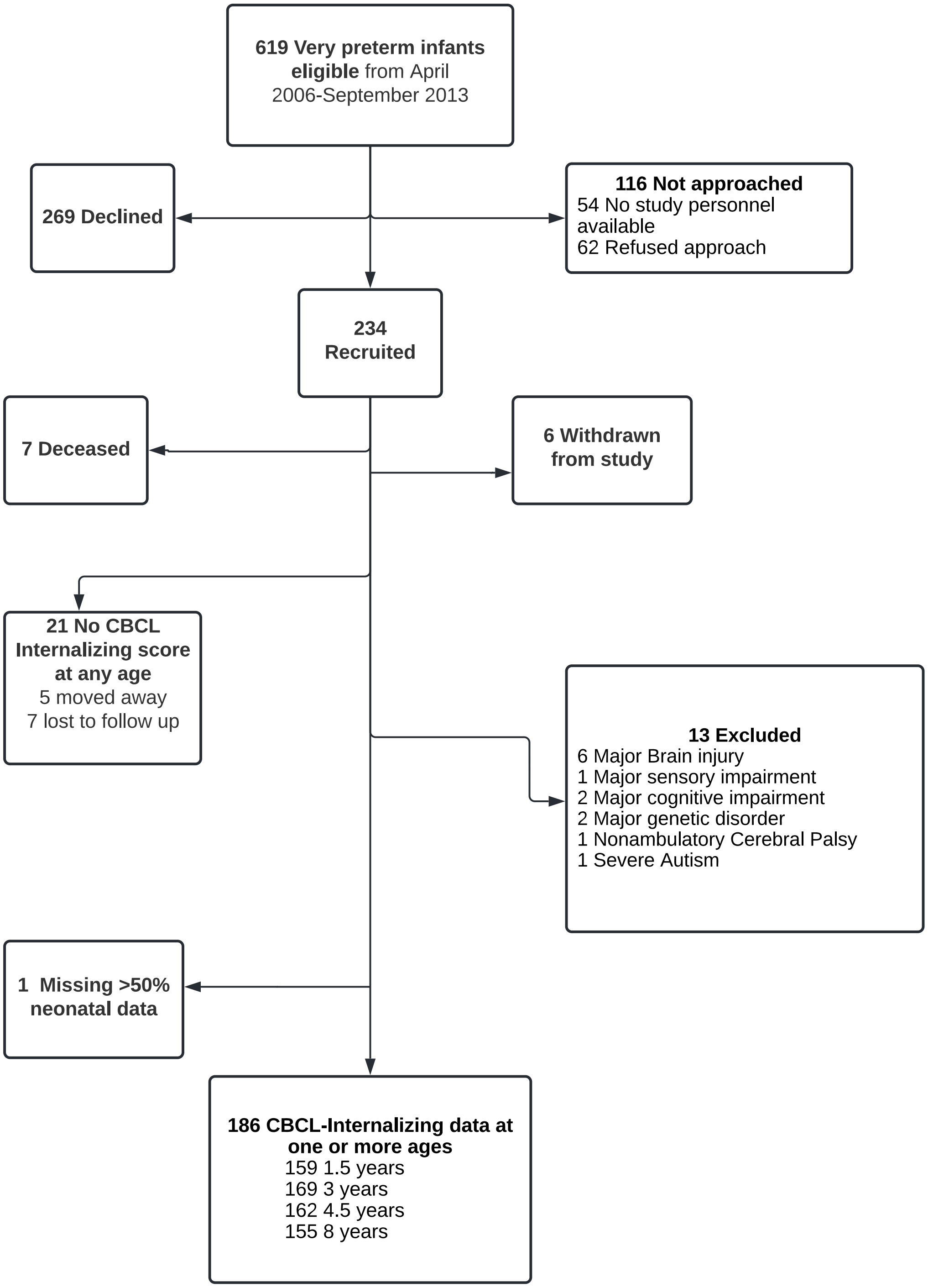

© 2022 McLean MA et al. *JAMA Network Open.*

eAppendix 1. Supplemental Methods

Study Population. Of the n = 227 survivors, 21 participants (6 withdrawn, 5 moved away, 7 lost to follow-up) did not have CBCL follow-up data at any age. We excluded 6 infants with severe brain injury on neonatal ultrasound (cystic periventricular leukomalacia [n = 2], intraventricular hemorrhage grade 3 or 4 [n = 4]); at age 4.5 and/or 8 years 1 sensori-neural hearing impairment, 1 non-ambulatory cerebral palsy, 2 cognitive impairment with IQ <70, 1 severe autism, 2 genetic abnormality (chromosome 15q11.2 duplication). Exclusion criterion were consistent with our work in a prior, independent cohort.[1] As well, one infant had incomplete (>50%) missing neonatal clinical data (moved hospital during NICU stay) and was therefore excluded.

Parent and Child Interactive Behaviors. At 1.5 and 3-years, the primary caregiver and child engaged in a 5-minute semi-structured teaching session.[1] During the session, the caregiver helped guide the child as they performed two tasks of varying difficulty. At 1.5 years, the easier and more familiar task consisted of the child stacking or nesting cups of varying sizes. In the second task, which was considered novel and more difficult, participants were directed to sort plastic pig and cow figurines into two separate bowls. At 3 years, the first task consisted of arranging a wooden puzzle of a family, while the more challenging task involved sorting plastic shapes by both colour and size. Parents were instructed to interact with their child typically as they would at home.

One primary and two reliability coders rated parent and child EA, blinded to all participant information. Inter-rater reliability assessed via intraclass correlation (ICC) on 20% of the sample was high: Parent Sensitivity, Structuring, Non-intrusiveness, and Non-hostility, and Child Responsiveness and Involvement at 18 months CA ICC's were 0.87, 0.86, 0.93, 0.84, 0.86, and 0.87 respectively, and at 3 years ICC's were 0.87, 0.85, 0.90, 0.83, 0.83, and 0.87.

© 2022 McLean MA et al. *JAMA Network Open.*

eAppendix 2. Statistical Analysis

Hypothesis 2: Latent profile analysis. Gaussian mixture modelling estimate model parameters via maximum likelihood estimation. We used the Bayes Information Criteria (BIC), Integrated Complete-data Likelihood (ICL), classification uncertainty, along with Bootstrapped Likelihood Ratio Test Statistic (LRTS) to determine the number of classes that best fit the data. Smaller values of BIC indicate better fit. LRT tests whether an increase in profiles increases fit. We examined plots for uncertainty, favouring models with fewer points of large (> 0.25) uncertainty. Conclusions as to the best fitting model were drawn from all three fit indices,[2] theoretical models of Emotional Availability,[3] and prior empirical work examining interrelationships between parent behavior, parenting stress and child behavior.[1,4]

Hypotheses 1 and 3: Multilevel models. Multilevel models examining the role of parent environment at 1.5 years used all data across 1.5, 3, 4.5, and 8 years. Subsequent models were run using behaviors from 3, 4.5 to 8 years when considering parenting behaviors and stress at age 3-years.

Multilevel models are ideal for analysis of repeated measures within observational study designs where data is missing at random.[5] For analysis with complete data, we fitted models using restricted maximum likelihood estimation which estimates parameters of analysis model by maximizing the observed-data likelihood. Model significance of effects was tested using the Satterthwaite approximations for degrees of freedom. These methods have been found to produce acceptable bias parameter estimates and Type 1 error rates even for smaller samples.[6–8] However, in multilevel models, missing data on the dependent variable is dealt with, while missing data on explanatory variables are not accounted for.[9] Using a joint modelling imputation approach, imputations were simultaneously generated from specified models and the clustered structure of the data was accounted for. This approach has been shown to produce less biased parameter estimates than other multiple imputation techniques particularly in the case of categorical and non-normally distributed longitudinal data collected at unevenly spaced time-points.[10]

Child age at visit (time-dependant measure) was specified as a first level variable and participant-specific measures (e.g. gestational age, neonatal pain-related stress, parent behavior, parenting stress) as second level variables. First an intercept-only model was established by allowing the intercept to enter as a random effect in order to examine the amount of variance in internalizing behaviors that is accounted for within and between individuals and to ensure the data required multi-level modelling analysis. Next, we tested whether internalizing behaviors were predicted by child age by adding this variable to the model (Model 1). We then examined whether the effect of age on internalizing behaviors differed between participants, entering age of the child as a random effect. If the random effect of age did not improve model fit, it was removed from the model prior to entering second-level variables. Next, second-level variables were entered into the model. First, we added gestational age at birth to confirm established reports of degree of prematurity in relation to internalizing behaviors (Model 2). Next, to test Hypothesis 1, we added clinical factors associated with prematurity, neonatal pain-related stress and child sex to the models (Model 3). We entered the interaction terms of neonatal pain-related stress and age into the model, and explored whether this interaction was subsequently dependant on child sex, or gestational age. To test Hypothesis 3, parent environment (parent behavior and parent stress) at each age were included as predictors in separate models, and subsequent models tested whether parenting factors interacted with neonatal pain-related stress in relation to internalizing behaviors across age (2-way interaction of Parenting X neonatal pain-related stress and 3-way interaction of Parenting X neonatal pain-related stress X Child Age). Finally, we tested whether effects were independent of child interactive behaviors and maternal education level (index of socioeconomic status) when examining relationships between parent interactive behaviors and child internalizing behaviors.

Reported models met all assumptions of multilevel models.

© 2022 McLean MA et al. *JAMA Network Open.*

eFigure 2. Patterns of Missing Data

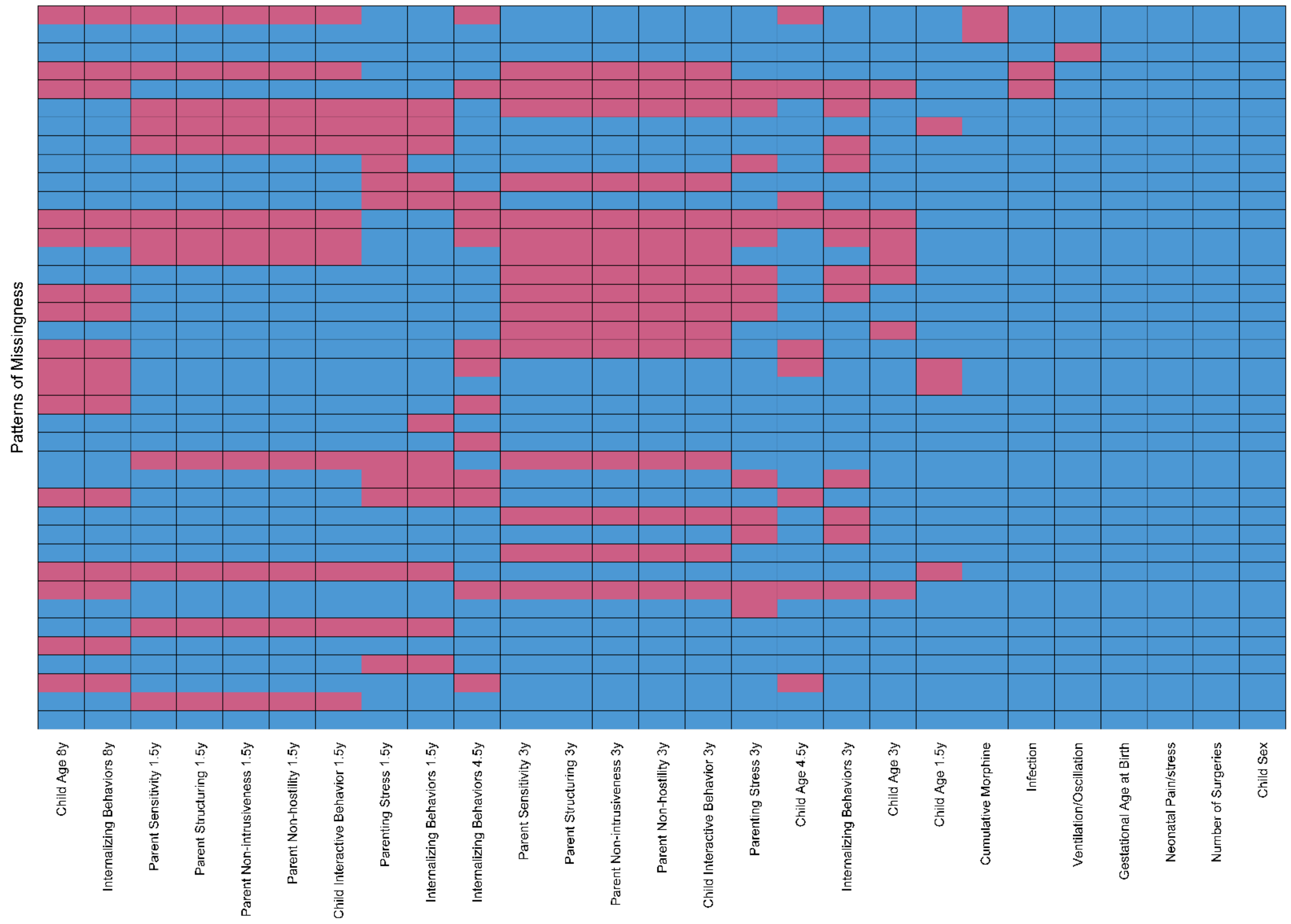

Note: Each row in the matrix represents a pattern of missing data in pink and complete data in blue, across the study variables (columns); 55.91% of participants had valid data across all study variables.

© 2022 McLean MA et al. *JAMA Network Open.*

eTable 1. Missing Data for Each Study Variable

Study Variable	Missing Data (%)
Child age 8y	18
Internalizing behaviors 8y	18
Parent sensitivity 1.5y	16
Parent structuring 1.5y	16
Parent non-intrusiveness 1.5y	16
Parent non-hostility 1.5y	16
Child interactive behavior 1.5y	16
Parenting stress 1.5y	15
Internalizing behaviors 1.5y	15
Internalizing behaviors 4.5y	13
Parent sensitivity 3y	11
Parent structuring 3y	11
Parent non-intrusiveness 3y	11
Parent non-hostility 3y	11
Child interactive behavior 3y	11
Parenting stress 3y	11
Child age 4.5y	1
Internalizing behaviors 3y	0.9
Child age 3y	0.5
Child age 1.5y	0.3
Cumulative morphine	0.1
Infection	0.1
Days on mechanical ventilation/oscillation	0.05
Gestational age at birth	0
Neonatal pain/stress	0
Number of surgeries	0
Child sex	0

© 2022 McLean MA et al. *JAMA Network Open.*

eAppendix 3. Supplemental Results

Intercorrelations

Children born at lower gestational age were exposed to higher neonatal cumulative pain/stress; *r* = -.78, *P*< 0.001. Lower gestational age at birth was associated with greater internalizing behaviors, with differences most detectable at ages 4 (*r* = -.16, *P* = .04) and 8 years (*r* = -18, *P* = .02).

© 2022 McLean MA et al. *JAMA Network Open.*

eTable 2. Intercorrelations Among CBCL Internalizing T-Scores, Parent and Child During Semistructured Teaching Task Within and Across Ages 1.5 and 3 Years

	Study Variable	1	P	2	P	3	P	4	P	5	P	6	P	7	P	8	P	9	P	10	P	11	P	12	P	13	P
**1**	Internalizing 1.5y																										
**2**	Internalizing 3y	.58	<.001																								
**3**	Parent sensitivity 1.5 y	.02	.78	-.05	.51																						
**4**	Parent structuring 1.5 y	.07	.38	.03	.75	.73	<.001																				
**5**	Parent non-intrusiveness 1.5 y	-.01	.88	-.1	.24	.85	<.001	.52	<.001																		
**6**	Parent non-hostility 1.5 y	.07	.38	-.05	.55	.92	<.001	.66	<.001	.80	<.001																
**7**	Child interactive behaviors 1.5 y	-.02	.85	-.11	.21	.71	<.001	.59	<.001	.65	<.001	.62	<.001														
**8**	Child interactive behaviors 3 y	-.14	.1	-.22	.005	.26	.002	.15	.08	.21	.08	.21	.01	.29	<.001												
**9**	Parent sensitivity 3 y	-.13	.12	-.2	.01	.31	<.001	.21	.01	.32	.01	.28	<.001	.23	.006	.74	<.001										
**10**	Parent structuring 3 y	-.09	.31	-.18	.03	.25	.003	.19	.03	.23	.03	.22	.009	.17	.04	.63	<.001	.79	<.001								
**11**	Parent non-intrusiveness 3 y	-.17	.04	-.28	<.001	.29	<.001	.16	.06	.31	.06	.26	.001	.20	.02	.68	<.001	.86	<.001	.63	<.001						
**12**	Parent non-hostility 3 y	-.08	.35	-.18	.02	.29	<.001	.19	.02	.29	.02	.26	.002	.21	.01	.67	<.001	.93	<.001	.76	<.001	.85	<.001				
**13**	Parenting stress 1.5 y	.36	<.001	.26	.001	.20	.02	.14	.09	.17	.09	.20	.02	.22	.009	.01	.87	-.03	.75	-.08	.35	.00	.98	-.04	.60		
**14**	Parenting stress 3 y	.23	.006	.36	<.001	.10	.23	.11	.21	.06	.21	.02	.83	.14	.10	-.05	.55	-.05	.50	-.10	.20	-.06	.45	-.02	.81	.78	<.001

© 2022 McLean MA et al. *JAMA Network Open.*

eTable 3. Demographic Comparison of Children With Complete Versus Incomplete Data

Study Variable	Complete data (n=103)	Incomplete data (n=83)	*P*
**Gestational age at birth (wks)**			.355
Mean (SD)	28.1 (2.4)	27.8 (2.0)	
Range (min - max)	24.0 - 32.3	24.4 - 31.7	
**Child sex, No. (%)**			.233
Boy	60 (58)	41 (49)	
**Maternal age at birth (years)**			.60
Mean (SD)	32.9 (5.3)	32.4 (5.3)	
Range (min - max)	22.1 - 45.9	21.8 - 45.1	
**Maternal marital status**			.81
Married/Common Law	95 (92)	73 (91)	
Single/Divorced/Separated	8 (8)	7 (9)	
**Maternal ethnicity, No. (%)**			.08
Black	0 (0)	0 (0)	
East/Southeast Asian	12 (11.5)	11 (13)	
East Indian	3 (3)	8 (10)	
Filipino	5 (5)	10 (12)	
First Nations	1 (1)	3 (3.5)	
White Caucasian	70 (68)	42 (51)	
Other[a]	11 (10.5)	6 (7)	
Did not report	1 (1)	3 (3.5)	
**Maternal education (years)**			.07
Mean (SD)	16.3 (2.9)	15.6 (2.8)	
Range (min - max)	10.0 - 23.0	10.0 - 27.0	
**Maternal Level of Education, No. (%)**			.1010
Primary or Secondary School Graduation	14 (14)	14 (18)	
Partial or Complete Undergraduate Degree	64 (63)	57 (71)	
Post-Graduate University degree	24 (24)	9 (11)	
**Paternal age at birth (years)**			.822
Mean (SD)	34.8 (5.7)	34.6 (5.7)	
Range (min - max)	22.0 - 48.7	20.9 - 48.9	
**Paternal marital status, No. (%)**			.044
Married/Common Law	84 (82)	53 (68)	
Single/Divorced/Separated	19 (18)	25 (32)	
**Paternal ethnicity, No. (%)**			.02
Black	0 (0)	1 (1)	
East/Southeast Asian	8 (8)	7 (8.5)	
East Indian	3 (3)	8 (10)	
Filipino	3 (3)	8 (10)	
First Nations	2 (2)	1 (1)	
White Caucasian	85 (82)	50 (60)	
Other[b]	2 (2)	1 (1)	
Did not report	0 (0)	7 (8.5)	
**Paternal education (years)**			.933
Mean (SD)	15.3 (2.8)	15.3 (3.5)	
Range (min - max)	10.0 - 23.0	9.0 - 27.0	
**Paternal level of education, no. (%)**			.733
Primary or Secondary School Graduation	22 (22)	20 (26)	
Partial or Complete Undergraduate Degree	66 (65)	45 (59)	
Post-Graduate University degree	14 (14)	11 (14)	

[a]*Latino, Melanesian, Metis, Mixed race;* [b]*Melanesian, Slavic*

© 2022 McLean MA et al. *JAMA Network Open.*

eTable 4. Final Models of Factors Related to Increased Child Internalizing Behaviors Across 3, 4.5, 8 Years Using Imputed Data

Study Variable	B (95% CI)			
	Model 1	Model 2	Model 3	Model 4
Child age at visit	1.29	1.28	1.28	1.28
	(0.98, 1.60)	(0.97, 1.59)	(0.98 1.59)	(0.97, 1.59)
Gestational age at birth	NA	-0.69	-0.61	-0.35
		(-1.28, -0.11)	(-1.72, 0.50)	(-1.42, 0.72)
Child sex[a]	NA	NA	1.82	1.99
			(-0.85, 4.50)	(-0.59, 4.57)
Illness severity (day 1)	NA	NA	0.10	0.09
			(-0.01, 0.21)	(-0.02, 0.20)
Neonatal pain-related stress	NA	NA	4.99	3.88
			(0.47, 9.50)	(-0.51, 8.27)
Cumulative morphine exposure	NA	NA	0.12	1.71
			(-4.62, 4.87)	(-2.98, 6.39)
Days on mechanical ventilation/oscillation	NA	NA	-0.72	-0.59
			(-1.63, 0.18)	(-1.47, 0.28)
Culture positive infection[b]	NA	NA	-2.39	-3.12
			(-6.04, 1.26)	(-6.65, 0.42)
Number of surgeries	NA	NA	0.14	0.04
			(-1.72, 2.00)	(-1.77, 1.85)
Parent environment 3 years[c]: Low Support, High Stress	NA	NA	NA	-5.47
				(-9.44, -1.51)
Parent environment 3 years[c]: High Support, Low Stress	NA	NA	NA	4.66
				(0.54, 8.77)
Child interactive behavior 3 years	NA	NA	NA	0.08
				(-0.19, 0.35)

Note: [a] Male = 0 [b] Confirmed infection = 1 [c] Reference group = Parent Environment 3 years: Average Support, Average Stress

© 2022 McLean MA et al. *JAMA Network Open.*

eTable 5. Estimated Multilevel Models of Internalizing Behaviors Across 1.5, 3, 4.5, 8 Years Using Listwise Deletion for Missing Data					
**Study Variable**	**B (95% CI)**				
	**Model 1**	**Model 2**	**Model 3**	**Model 4**	**Model 5**
Child age at visit	1.29	1.29	1.28	1.30	1.37
	(1.06, 1.52)	(1.06, 1.52)	(1.05, 1.52)	(1.05, 1.56)	(1.12, 1.62)
Gestational age at birth	NA	-0.63	-0.40	-1.04	-0.24
		(-1.17, -0.09)	(-1.46, 0.66)	(-2.16, 0.08)	(-1.27, 0.79)
Child sex[a]	NA	NA	2.21	1.21	0.63
			(-0.33, 4.75)	(-1.41, 3.82)	(-1.93, 3.18)
Illness severity (day 1)	NA	NA	0.09	0.12	0.07
			(-0.01, 0.20)	(0.01, 0.22)	(-0.03, 0.18)
Neonatal pain-related stress	NA	NA	4.82	2.14	3.50
			(0.53, 9.10)	(-2.44, 6.71)	(-0.63, 7.63)
Cumulative morphine exposure	NA	NA	-0.64	-2.98	1.01
			(-5.22, 3.93)	(-7.79, 1.84)	(-3.52, 5.53)
Days on mechanical ventilation/oscillation	NA	NA	-0.71	-0.96	-0.57
			(-1.58, 0.16)	(-1.98, 0.05)	(-1.41, 0.28)
Culture positive infection[b]	NA	NA	-1.33	-1.41	-1.94
			(-4.74, 2.08)	(-4.90, 2.08)	(-5.32, 1.44)
Number of surgeries	NA	NA	0.16	0.88	0.16
			(-1.63, 1.94)	(-0.97, 2.73)	(-1.58, 1.89)
Parenting stress 1.5 years	NA	NA	NA	0.17	NA
				(0.10, 0.24)	
Parent environment 1.5 years[e]: Low Support	NA	NA	NA	-0.06	NA
				(-3.89, 3.76)	
Parent environment 1.5 years[e]: High Support	NA	NA	NA	1.28	NA
				(-4.18, 6.73)	
Child interactive behavior 1.5 years	NA	NA	NA	-0.29	NA
				(-0.56, -0.01)	
Parent environment 3 years[d]: Low Support, High Stress	NA	NA	NA	NA	4.92
					(1.15, 8.69)
Parent environment 3 years[d]: High Support, Low Stress	NA	NA	NA	NA	-5.31

© 2022 McLean MA et al. *JAMA Network Open.*

					(-8.97, -1.66)
Child interactive behavior 3 years	NA	NA	NA	NA	0.06
					(-0.19, 0.31)
Maternal education (years)					-0.50
	NA	NA	NA	NA	(-0.97, -0.04)
Note: [a] Male = 0, [b] Confirmed infection = 1, [c] Reference group = Parent Environment 1.5 years: Average Support, [d] Reference group = Parent Environment 3 years: Average Support, Average Stress					

© 2022 McLean MA et al. *JAMA Network Open.*

eTable 6. Estimated Multilevel Models of Internalizing Behaviors Across 3, 4.5, 8 Years Using Listwise Deletion for Missing Data				
**Study Variable**	**B (95% CI)**			
	**Model 1**	**Model 2**	**Model 3**	**Model 4**
Child age at visit	1.28	1.28	1.27	1.31
	(0.97, 1.59)	(0.97, 1.59)	(0.96, 1.58)	(0.99, 1.64)
Gestational age at birth	NA	-0.69	-0.74	-0.53
		(-1.28, -0.11)	(-1.85, 0.37)	(-1.65, 0.58)
Child sex [a]	NA	NA	1.55	0.54
			(-1.11, 4.20)	(-2.22, 3.29)
Illness severity (day 1)	NA	NA	0.11	0.10
			(-0.01, 0.22)	(-0.01, 0.21)
Neonatal pain-related stress	NA	NA	4.66	3.45
			(0.13, 9.19)	(-1.02, 7.91)
Cumulative morphine exposure	NA	NA	0.12	1.66
			(-4.62, 4.87)	(-3.21, 6.53)
Days on mechanical ventilation/oscillation	NA	NA	-0.72	-0.68
			(-1.63, 0.18)	(-1.59, 0.22)
Culture positive infection [b]	NA	NA	-2.39	-3.34
			(-6.04, 1.26)	(-6.99, 0.32)
Number of surgeries	NA	NA	0.14	0.22
			(-1.72, 2.00)	(-1.65, 2.08)
Parent environment 3 years [c]: Low Support, High Stress	NA	NA	NA	-5.62
				(-9.56, -1.68)
Parent environment 3 years [c]: High Support, Low Stress	NA	NA	NA	4.88
				(0.82, 8.95)
Child interactive behavior 3 years	NA	NA	NA	0.07
				(-0.20, 0.34)
Maternal education (years)	NA	NA	NA	-0.57
				(-1.07, -0.07)
Note: [a] Male = 0 [b] Confirmed infection = 1 [c] Reference group = Parent Environment 3 years: Average Support, Average Stress				

eReferences

1. Vinall J, Miller SP, Synnes AR, Grunau RE. Parent behaviors moderate the relationship between neonatal pain and internalizing behaviors at 18 months corrected age in children born very prematurely: *Pain*. 2013;154(9):1831-1839. doi:10.1016/j.pain.2013.05.050

2. Nylund KL, Asparouhov T, Muthén BO. Deciding on the Number of Classes in Latent Class Analysis and Growth Mixture Modeling: A Monte Carlo Simulation Study. *Structural Equation Modeling: A Multidisciplinary Journal*. 2007;14(4):535-569. doi:10.1080/10705510701575396

3. Biringen Z. Emotional availability: Conceptualization and research findings. *American Journal of Orthopsychiatry*. 2000;70(1):104-114. doi:10.1037/h0087711

© 2022 McLean MA et al. *JAMA Network Open.*

4. Tu MT, Grunau RE, Petrie-Thomas J, Haley DW, Weinberg J, Whitfield MF. Maternal stress and behavior modulate relationships between neonatal stress, attention, and basal cortisol at 8 months in preterm infants. *Developmental Psychobiology*. 2007;49(2):150-164. doi:10.1002/dev.20204

5. Detry MA, Ma Y. Analyzing Repeated Measurements Using Mixed Models. *JAMA*. 2016;315(4):407-408. doi:10.1001/jama.2015.19394

6. Luke SG. Evaluating significance in linear mixed-effects models in R. *Behav Res*. 2017;49(4):1494-1502. doi:10.3758/s13428-016-0809-y

7. Shin T, Davison ML, Long JD. Maximum likelihood versus multiple imputation for missing data in small longitudinal samples with nonnormality. *Psychological Methods*. 2017;22(3):426-449. doi:10.1037/met0000094

8. Messer K, Natarajan L. Maximum likelihood, multiple imputation and regression calibration for measurement error adjustment. *Stat Med*. 2008;27(30):6332-6350. doi:10.1002/sim.3458

9. Grund S, Lüdtke O, Robitzsch A. Multiple Imputation of Missing Data for Multilevel Models: Simulations and Recommendations. *Organizational Research Methods*. 2018;21(1):111-149. doi:10.1177/1094428117703686

10. Huque MH, Carlin JB, Simpson JA, Lee KJ. A comparison of multiple imputation methods for missing data in longitudinal studies. *BMC Medical Research Methodology*. 2018;18(1):168. doi:10.1186/s12874-018-0615-6

© 2022 McLean MA et al. *JAMA Network Open.*
